# Clinical learning environments across two different healthcare settings using the undergraduate clinical education environment measure

**DOI:** 10.1186/s12909-023-04467-y

**Published:** 2023-07-05

**Authors:** Hani TS Benamer, Laila Alsuwaidi, Nusrat Khan, Lisa Jackson, Jeyaseelan Lakshmanan, Samuel B. Ho, Catherine Kellett, Alawi Alsheikh-Ali, Adrian G Stanley

**Affiliations:** 1 College of Medicine, Mohammed Bin Rashid University of Medicine and Health Sciences, United Arab Emirates Building 14, Dubai Healthcare City, Dubai, PO Box 505055, UAE; 2 Brunel Medical School, College of Health, Medicine and Life Sciences, Brunel University London, London, UK

**Keywords:** Clinical learning environments, Undergraduate Clinical Education Environment measure, Undergraduate medical students, Private Health Sector, Public Health Sector

## Abstract

**Background:**

The clinical placements of our medical students are almost equally distributed across private and public sectors. This study aims to assess medical students’ perceptions of their Clinical learning Environment (CLE) across these two different healthcare settings, using the Undergraduate Clinical Education Environment Measure (UCEEM).

**Methods:**

76 undergraduate medical students (Year 5 and 6), were invited to participate. Data were collected using an online UCEEM with additional questions related to demographics and case load exposure. The UCEEM consists of two overarching domains of experiential learning and social participation, with four subdomains of learning opportunities, preparedness, workplace interaction, and inclusion.

**Results:**

38 questionnaires were received. Of 225 responses to the individual UCEEM items, 51 (22.6%) scored a mean of ≥ 4 (range 4-4.5, representing strong areas), 31 (13.7%) scored a mean of ≤ 3 (range 2.1-3, needing attention) and 143 (63.6%) scored a mean of 3.1–3.9 (areas that could be improved). The majority (63%) of the case load exposure responses scored a mean of ≥ 4 (range 4-4.5). Compared to the private sittings, there is a significant reduction in total UCEEM (p = 0.008), preparedness for student entry (p = 0.003), and overarching dimension of social participation (p = 0.000) scores for the public sector. Similarly, both workplace interaction patterns and student inclusion and equal treatment scored significantly lower for the public sector (p = 0.000 and p = 0.011 respectively). Two out of three case load exposure items scored significantly higher for the public sector (p = 0.000).

**Discussion:**

The students’ CLE perceptions were generally positive. The lower UCEEM ratings in the public sector items were related to student entry preparedness, workplace interactions, student inclusiveness and workforce equity of treatment. In contrast the students were exposed to more variety and larger number of patients in the public sector. These differences indicated some significantly different learning environments between the two sectors.

## Introduction

Spencer defined clinical teaching as *“teaching and learning focused on, and usually directly involving patients and their problems*” [1]. The clinical learning environment (CLE) consists primarily of hospitals and primary/community healthcare centres [2]. Medical students learn their clinical skills in rather complex clinical environments by rotating through different clinical clerkships [3, 4]. Each CLE has its own distinct features, culture and values [2] and provide the students with the opportunities to learn from role modelling and the hidden curriculum. Therefore, the CLE is an educational environment vital for successful learning [5] and evaluating it is essential.

In their systematic review of the instruments to assess healthcare educational environments, Soemantri et al. concluded that reliable and validated tools are available to measure the learning environment and thus this should be the normal practice in any medical school [6]. The Dundee Ready Education Environment Measure (DREEM) is widely used [6], but is not specifically designed to assess the CLE [7]. The Undergraduate Clinical Education Environment Measure (UCEEM) was created and validated to assess CLE [7, 8]. The UCEEM was first developed in Sweden [6, 9] and assesses students’ perception of their learning experience, social participation and pedagogical quality of CLE [7, 8, 10].

The United Arab Emirates (UAE) is a constitutional federation of seven emirates and has a population of ~ 9.5 million of which approximately 70% live in the emirates of Abu Dhabi and Dubai [11]. Around 85% of the population are expatriates [11]. Since formation in 1971, the UAE leadership have invested oil revenues to accelerate the growth of the finance, healthcare, and education sectors. Consequently, the UAE population has experienced significant improvements in health and wealth [11, 12].There are eight medical schools in the UAE [13]. The various medical programmes must be approved by the Ministry of Education and accredited by the Commission for Academic Accreditation [13]. All but one of the programmes are 5–6 years long, taking students from secondary schools [13].The College of Medicine in Mohammed Bin Rashid University of Medicine and Health Sciences (MBRU), in Dubai, was inaugurated in 2016 and its first cohort graduated in June 2022. The MBRU offer a 6-year MBBS course which is divided into three phases. Phase 1 (Year 1) covers the fundamental concepts in science. Phase 2 (Years 2–3) includes systems (e.g., neurosciences) and integrated medicine courses. In phase 3, (Years 4–6), the students rotate through a series of clinical clerkships, the final year of which is based on an internship-style programme.

Distinctively, our medical students undertake their clinical clerkships in hospitals and community clinics in both the private and public sectors. This unique setting exposes the students to two, potentially, different sets of cultures and clinical environments. Thus, this study is aiming to assess undergraduate medical students’ perceptions of their CLE in general and across two different healthcare settings using the UCEEM. To the best of our knowledge, this is the first study to use the UCEEM in the UAE.

## Methods

### Study design and data collection

This questionnaire-based, cross-sectional, study used the UCEEM with three additional questions on demographics and three questions to assess student caseload exposure; the latter mindful of early student evaluation suggesting some differences.

All Year 5 and 6 MBBS students were invited to participate anonymously in the study and reflect on all placements they had experienced, to date, in Phase 3. By completing two UCEEM questionnaire, students were asked to provide answers based on their placements in public hospitals/clinics on one form and private hospitals/clinics on the other. Two reminders were sent: 4 and 6 weeks after the first invitation.

The study was approved by MBRU Ethical Review Board, (MBRU IRB-2021-90).

### UCEEM data analysis

The UCEEM consists of two overarching domains of experiential learning and social participation, with four subdomains of learning opportunities, preparedness, workplace interaction, and inclusion. Items/questions were scored on a five-point Likert scale (ranging from 1 = fully disagree to 5 = fully agree, a higher score indicates a more positive score) [8] and analysed at individual (25 items/questions), four subdomains, and two overarching domains levels (Table 1). Details about the UCEEM items and psychometric features have already been reported [7, 8].

**Table 1:** The scores for the UCEEM (total, the scales, and the items) and the extra questions for all students

UCEEM overarching dimensions, subsets, and items		Public health sector mean (SD)				Private health sector mean (SD)				Public & private health sectors mean (SD)				P value		
		**All** **n = 21**	**Year 6** **n = 10**	**Year 5** **n = 11**		**All** **n = 17**	**Year 6** **n = 7**	**Year 5** **n = 10**		**All** **n = 38**	**Year 6** **n = 17**	**Year 5** **n = 21**		**Public health sector = year 6 vs. year 5**	**Private health sector = year 6 vs. year 5**	**Public vs. private health sectors = all students**
Subset A1: Opportunities to learn in and through work and quality of supervision																
3. My (work) tasks are relevant to the learning objectives		3.9(0.8)	3.7(0.9)	4.1(0.7)		3.8(0.9)	3.7(0.8)	3.9(1.0)		3.9(0.8)	3.7(0.8)	4.0(0.8)		0.267	0.618	0.719
4. I am sufficiently occupied with meaningful (work) tasks		3.6(0.9)	3.7(0.9)	3.5(0.8)		3.4(1.1)	3.0(0.8)	3.6(1.2)		3.5(1.0)	3.4(0.9)	3.5(1.0)		0.596	0.268	0.541
5. My tasks are suitably challenging for my level of knowledge and skills		3.6(0.9)	3.7(0.8)	3.5(0.9)		3.3(1.2)	2.9(1.2)	3.6(1.1)		3.5(1.0)	3.4(1.1)	3.5(1.0)		0.598	0.233	0.384
6. I am encouraged to participate actively in the work here		3.6(1.1)	3.7(1.1)	3.5(1.1)		3.5(1.1)	3.0(1.2)	3.9(1.0)		3.3(1.1)	3.4(1.2)	3.7(1.1)		0.682	0.112	0.782
13. I receive useful feedback from my supervisors		3.1(1.1)	2.7(1.2)	3.5(0.9)		3.7(1.0)	3.4(1.3)	3.9(1.0)		3.4(1.1)	3.0(1.2)	3.7(1.0)		0.098	0.383	0.090
14. I feel able to ask my supervisors any question I wish.		3.8(1.1)	3.2(1.0)	4.3(0.9)		4.2(0.5)	3.9(0.3)	4.4(0.5)		4.0(0.9)	3.5(0.9)	4.3(0.7)		0.015*	0.032*	0.174
15. I get the opportunity to provide a rationale for my actions during supervision sessions		3.2(1.0)	2.9(1.0)	3.5(0.9)		3.8(0.7)	3.6(0.5)	4.0(0.8)		3.5(1.0)	3.2(0.9)	3.8(0.9)		0.164	0.261	0.043*
16. My problem-solving skills are developing well in this placement		4.0(0.6)	3.9(0.3)	4.2(0.8)		3.8(0.7)	3.7(0.5)	3.9(0.9)		4.0(0.7)	3.8(0.4)	4.0(0.8)		0.278	0.603	0.349
17. I have the opportunity to put my theoretical knowledge into practice in this placement		4.1(0.7)	4.0(0.8)	4.3(0.6)		3.9(0.7)	3.9(0.4)	4.0(0.9)		4.0(0.7)	3.9(0.7)	4.1(0.8)		0.340	0.788	0.387
18. I have the opportunity to learn together with other medical students in this placement		4.1(0.8)	4.3(0.7)	4(0.9)		3.4(0.9)	3.9(0.7)	3.1(1.0)		3.8(0.9)	4.1(0.7)	3.6(1.2)		0.408	0.088	0.015*
25. I feel I have influence over my learning in this placement		3.7(0.9)	3.4(1.1)	3.9(0.7)		4.0(0.7)	3.7(0.8)	4.2(0.6)		3.8(0.8)	3.5(0.9)	4.0(0.7)		0.224	0.160	0.267
Total; Subset A1 (Total = 55)		40.7	39.2	42.3		40.8	38.7	42.5		40.8	38.9	42.2		0.121	0.038*	0.797
Subset A2: Preparedness for student entry																
1. I received useful induction in this placement		3.0(1.2)	2.4(1.3)	3.6(0.9)		3.8(1.1)	3.3(1.1)	4.1(1.0)		3.4(1.2)	2.8(1.3)	3.9(1.0)		0.022*	0.139	0.041*
2. My supervisors were expecting me when I arrived.		3.1(1.2)	2.7(1.3)	3.5(1.0)		4.2(0.7)	4.3(0.5)	4.1(0.9)		3.6(1.6)	3.3(1.3)	3.8(1.0)		0.128	0.603	0.001*
9. I have a supervisor to whom I know I can turn		3.6(0.9)	3.4(1.0)	3.7(0.9)		4.1(0.9)	3.9(0.9)	4.2(0.9)		3.8(0.9)	3.6(0.9)	4.0(0.9)		0.478	0.509	0.097
10. I have sufficient access to supervision		3.5(0.7)	3.4(0.7)	3.5(0.8)		4.4(0.6)	4.1(0.7)	4.5(0.5)		3.9(0.8)	3.7(0.8)	4.0(0.8)		0.764	0.187	0.000*
11. The supervisors are well prepared for supervising		3.2(1.0)	2.7(0.9)	3.7(0.9)		3.9(1.0)	3.4(0.5)	4.3(1.1)		3.6(1.1)	3.0(0.9)	4.0(1.0)		0.019*	0.062*	0.038*
12. It is clear that my supervisors are familiar with the learning objectives		2.7(1.1)	2.4(1.1)	2.9(1.0)		3.6(1.1)	3.4(0.8)	3.7(1.3)		3.1(1.2)	2.8(1.1)	3.3(1.2)		0.288	0.597	0.016*
Total; Subset A2 (Total = 30)		19.1	17	20.9		24	22.4	24.9		21.4	19.2	23		0.015*	0.060	0.003*
Total; Overarching dimension experiential learning (A1 + A2 Total = 85)		59.8	56.2	63.2		64.8	61.1	67.4		62.2	58.1	65.2		0.018*	0.005*	0.119
Subset B1: Workplace interaction patterns and student inclusion																
7. I have adequate access to computers in this placement		2.3(1.3)	2.5(1.3)	2.1(1.4)		4.1(1.0)	4.0(1.2)	4.2(0.9)		3.1(1.5)	3.1(1.4)	3.1(1.6)		0.507	0.691	0.000*
8. There is sufficient physical space for the number of medical students on placement here		2.5(1.4)	2.4(1.4)	2.5(1.4)		4.5(0.5)	4.4(0.5)	4.5(0.5)		3.4(1.5)	3.2(1.5)	3.5(1.4)		0.871	0.684	0.000*
19. As a student I am received in a positive way by the staff here		3.2(1.7)	2.7(1.6)	3.6(1.2)		4.1(0.8)	4.0(0.6)	4.1(1.0)		3.6(1.1)	3.2(1.1)	3.9(1.0)		0.158	0.486	0.052
20. I feel included in the team of people who work here		3.4(1.2)	3.4(1.3)	3.4(1.1)		3.7(0.9)	3.7(0.8)	3.7(1.1)		3.5(1.1)	3.5(1.1)	3.5(1.1)		1.000	1.000	0.398
21. Communication between those working here is good		3.0(1.8)	2.9(0.9)	3.2(1.3)		3.8(0.9)	3.6(1.0)	3.9(0.9)		3.4(1.1)	3.2(1.0)	3.5(1.1)		0.550	0.518	0.133
24. I feel welcome in the staff room/lunchroom here		3.2(1.1)	2.7(1.1)	3.6(1.0)		3.6(1.1)	3.4(1.4)	3.8(0.9)		3.4(1.1)	3.0(1.2)	3.7(1.0)		0.064	0.468	0.272
Total; subset B1 (Total = 30)		17.6	16.6	18.4		23.8	23.1	24.2		20.4	19.2	21.2		0.335	0.362	0.000*
Subset B2: Equal treatment																
22. Everyone is treated equally here regardless of cultural background		3.2(1.4)	2.5(1.4)	3.9(1.3)		4.1(1.0)	3.7(1.3)	4.4(0.7)		3.6(1.3)	3.0(1.5)	4.1(1.0)		0.028*	0.710	0.032*
23. Everyone is treated equally here regardless of gender		3.2(1.3)	2.5(1.3)	3.8(1.0)		4.1(1.0)	3.6(1.2)	4.5(0.5)		3.6(1.2)	2.9(1.3)	4.1(0.9)		0.018*	0.058	0.024*
Total; subset B2 (Total = 10)		6.4	5	7.7		8.2	7.3	8.9		7.2	5.9	8.2		0.001*	0.007*	0.011*
Total; overarching dimension social participation (B1 + B2 Total = 40)		24	21.6	26.1		32	30.4	33.1		27.6	25.1	29.4		0.068	0.051	0.000*
Total score/125 (UCEEM)		83.8	77.8	89.3		96.8	91.5	100.5		89.8	83.2	94.6		0.004*	0.009*	0.008*
Extra questions related to case load exposure																
A. I have been exposed to variety of patients during my placement		4.4(0.7)	4.5(0.5)	4.4(0.9)		3.3(1.0)	3.3(1.0)	3.3(1.2)		3.9(1.0)	4.0(0.9)	3.9(1.2)		0.759	1.000	0.000*
B. I have been exposed to sufficient number of patients during my placement		4.5(0.5)	4.5(0.5)	4.5(0.5)		3.5(1.1)	3.3(1.1)	3.6(1.1)		4.0(0.9)	4.0(1.0)	4.0(0.9)		1.000	0.588	0.000*
C. I was practically involved (taking history and/or performing physical examination) in sufficient number of patients during my placement		4.2(0.8)	4.4(0.8)	4.1(0.7)		3.9(0.7)	3.9(0.7)	4.0(0.7)		4.1(0.7)	4.2(0.8)	4.0(0.7)		0.370	0.775	0.232
Total score/15		13.1	13.4	13		10.7	10.5	10.9		12	12.2	11.9		0.766	0.672	0.001*

Abbreviations; n = number of the students; UCEEM = Undergraduate clinical education environment measure. * *P*-value of less than 0.05 (statistically significant).

Scores represent five-point Likert scale (ranging from 1 = fully disagree to 5 = fully agree) Mean score interpretation: ≥4 = strong areas, ≤ 3 = needs attention, 3.1–3.9 = areas for improvement.

Items/questions were reported as mean scores: those with a score of ≥ 4 were considered as strong areas, those with a score ≤ 3 as needing attention and those with a score 3.1–3.9 as areas that could be improved. This classification was a modification of a previous study [7].

The paired t-test was used to compare the results of each item (private vs. public healthcare setting and Year 5 vs. Year 6 within each sector). Regression coefficient analysis was conducted to compare the subset, overall levels and the sum of the extra questions. Statistical significance was set at *P*-value < 0.05. All statistical analyses were performed using Excel and SPSS. We acknowledge that Likert scale data are ordinal, but we used the relevant statistical tests based on previous published UCEEM studies [7, 10].

## Results

### Participants

76 students were eligible to participate (Year 5: 34 students; Year 6: 42 students). Out of potential 152, 38 questionnaires were submitted, (21 related to the public and 17 to the private sector) which represents an overall rater response of 25%. All the 38 questionnaires were submitted by students aged 21–24 (except 4 questionnaires where the submitting students were older). 68% of the questionnaires were submitted by female students which reflects the gender distribution of the student cohorts.

### UCEEM individual items (Table 1)

Out of 225 responses to the individual UCEEM items, 51 (22.6%) scored a mean of ≥ 4 (range 4-4.5, representing strong areas), 31 (13.7%) scored a mean of ≤ 3 (range 2.1-3, needing attention) and 143 (63.6%) scored a mean of 3.1–3.9 (areas that could be improved). The majority (63%) of the case load exposure responses scored a mean of ≥ 4 (range 4-4.5) and there was no response scored a mean of less than 3.3 (Table 1).

### UCEEM score comparing private and public sittings (Table 1)

Compared to the private sittings, and after adjusting for cohort year and student gender, public setting had significantly lower scores in total of the UCEEM (p = 0.008), preparedness for student entry (placement orientation) (subset A2) (p = 0.003), and the overarching dimension of social participation (total subset of B1 and B2) (p = 0.000). Similarly, both workplace interaction patterns and student inclusion (subset B1) and equal treatment (B2) scored significantly lower for the public sector (p = 0.000 and p = 0.011 respectively).

11 individual UCEEM items revealed a statistically significant difference. Nine were in subsets A2, B1 and B2 and revealed a lower score for the public sector which is consistent with earlier findings. Two were in subset A1: a lower score for the public sector was noted for getting the opportunity to provide a rationale for actions during supervision sessions; yet a higher score was achieved in the public sector for the opportunity to learn together with other medical students. The latter reflects the private setting’s exclusive access for MBRU undergraduate medical students.

### UCEEM score comparing year 5 and Year 6 students (Table 1)

In the public sector, scores by Year 5 students were significantly higher than scores by Year 6 students in UCEEM items: 1, 11, 14, 22, 23, subset A2, subset B2, overarching dimension experiential learning (A1 + A2), and total UCEEM. In the private sector, scores by Year 5 students were significantly higher than scores by year 6 students in UCEEM items: 11, 14, subset A1, subset B2, overarching dimension experiential learning (A1 + A2), and total UCEEM.

### Extra questions related to case load exposure (Table 1)

Two out of three extra items relating to (A) variety of cases and (B) exposure to a high number of cases scored significantly higher for the public sector (p = 0.000). The total score of the three extra items was significantly higher for the public sector (p = 0.001). There were no differences between Year 5 and Year 6 students.

## Discussion

In our study, only 14% of the UCEEM items scored ≤ 3 which represents area needing attention and around 23% of items scored ≥ 4 and thus representing strong areas. The rest revealed areas that could be improved. This indicates that students’ CLE perceptions were generally positive. However, there were statistically significant differences between the public and private sectors in many items. This requires further evaluation as it is essential to create a strongly positive learning environment and thus a good quality of life for medical students [14].

It is not possible to directly compare our results with other schools in the Gulf region as our study is the first to use the UCEEM. A study from Gulf Medical College (UAE) revealed a positive educational environment among medical students using DREEM (120/200) [15], whilst a study from Saudi Arabia reported a lower DREEM score (102/200) [16]. UCEEM was used to assess medical student perceptions at the Karolinska Institute, Sweden and Aberdeen, UK [7, 10]. Our total UCEEM score (89.8) sits between the Swedish (87) [7] and the UK (93.5) [10] schools. In addition to the differences between public and private sectors, total UCEEM score was significantly higher in Year 5 (94.6) compared to Year 6 students (83.2). Therefore, our results are comparable with the available international data, albeit with a lower sample size (n = 38) compared to studies from Sweden (n = 128) [7] and UK (n = 132) [10]. These studies did not compare private vs. public sector hospitals, and further studies examining learning environments in these two different settings are needed.

The lower UCEEM ratings in the public sector focussed mostly on items related to student entry preparedness (placement orientation), workplace interactions, student inclusiveness and workforce equity of treatment. In the public, compared to the private sector, students perceived that the supervisor was accessible but not well prepared. Moreover, they perceived a less welcoming environment in the workplace. Students also perceived that they were not treated equally regardless of their gender or cultural background, when compared to the private sector. Generally, these differences were more apparent to Year 6 students. On the other hand, the public sector scored significantly higher in 2 out of the 3 extra questions, which indicate that the medical students felt that they were exposed to a larger number and more variety of patients.

These findings indicate that the learning opportunities for students and quality of supervision is satisfactory and similar in both private and public health sectors. In the public sector, the patient case-mix is more varied and in greater number. Anecdotally, patients presenting to the private sector are younger, less likely to have co-morbidities and present earlier in their illness. Maybe the pool of potential patients accessing private healthcare is also limited to those with an acceptable health insurance and/or ability to pay. On the contrary, the demands of a busy clinical service in the public sector may explain why some aspects of the student entry preparedness, workplace interactions and student inclusiveness were perceived less favourably. There may be less time for medical and non-medical staff to engage with students which they contrast with the ‘luxury’ afforded to them by private healthcare workers. In the public sector, there may be a greater willingness to allow students to ‘jump’ into action with little preparation.

The students perceived that healthcare workers were not treated equally (cultural background and gender) in the public compared to private sectors. This score (72%) was much lower than the Swedish (83%) [7] or UK (90%) [10] school. This was most evident in Year 6 students, but a trend was also noted in Year 5. For the Year 6 students, the response was almost bimodal: nine (of 15) students rated both items as ≥ 4, while five rated the same ≤ 2. We do not know if there was a specific concern noted in one or two specialties and clearly this finding will need further analysis.

The differences seen between Year 5 and Year 6 students are most likely explained by the nature of their clinical placements. The Year 5 placements are more structured and typical of a traditional clinical clerkship. In Year 6, students are attached to a specialty team and undertake an internship-style programme: there is a greater expectation that they work with the team to develop their skills. Thus, students are likely to perceive that the placements are not as well-structured but will have a greater insight into student-staff and staff-staff interactions.

This study has several limitations. The students’ responses are based on their perceptions and have limitations. The cross-sectional study design can lead, as Roberts describes, to a “snapshot” view [10]. Moreover, the student number is low, which may cause a response bias. However, the study still generated statistically significant outcomes. Like many clerkship programmes, the students rotate to different hospitals within each health sector, thus they do not have identical experiences. Nevertheless, we endeavour to ensure equity in experience for all students. We asked students to reflect on the totality of their experiences in two questionnaires. This does not discriminate between different institutions and therefore subtle variations at a hospital level cannot be deciphered. However, the alternative approach of requesting multiple questionnaires would challenge even our most patient students. Finally, the study was not designed to explore detailed reasons for any unexpected findings, e.g., specific UCEEM items scoring poorly. Further evaluation by using a focus group approach or triangulation with student evaluation could help. We are planning to conduct the same analysis in subsequent academic years to overcome some of the above-mentioned limitations and assess the effectiveness of any intervention to enhance the CLE.

In summary, this is the first study to use the UCEEM in the UAE and Gulf region and compare private and public healthcare sectors. Generally, the study indicates a positive student CLEs perception. Our study showed that the students’ perception to different components of the UCEEM favour the private healthcare sector settings in social participation and preparedness for student entry, which may reflect that achieving the balance between delivering teaching and service in the public sector may be more difficult. There was no difference between the two settings regarding opportunities to learn and quality of supervision. In contrast the students were exposed to more variety and larger number of patients in the public sector, which may reflect the large number of patients who are using the public sector. Nevertheless, the unique exposure of the students to two different healthcare settings, reflective of healthcare delivery in the UAE, allows for a comprehensive, complementary real-life experience. Effective interventions are needed to optimise the learning and social participation experience of our students and further longitudinal studies are required to evaluate the impact of any intervention.

## Data Availability

The datasets used and/or analysed during the current study available from the corresponding author on reasonable request.
